# 5-HTTLPR Polymorphism Impacts Task-Evoked and Resting-State Activities of the Amygdala in Han Chinese

**DOI:** 10.1371/journal.pone.0036513

**Published:** 2012-05-04

**Authors:** Sufang Li, Qihong Zou, Jun Li, Jin Li, Deyi Wang, Chaogan Yan, Qi Dong, Yu-Feng Zang

**Affiliations:** 1 State Key Laboratory of Cognitive Neuroscience and Learning, Beijing Normal University, Beijing, China; 2 MRI Research Center and Beijing City Key Lab for Medical Physics and Engineering, Peking University, Beijing, China; 3 Center for Cognition and Brain Disorders, Affiliated Hospital, Hangzhou Normal University, Hangzhou, Zhejiang, China; French National Centre for Scientific Research, France

## Abstract

**Background:**

Prior research has shown that the amygdala of carriers of the short allele (s) of the serotonin transporter (5-HTT) gene (5-HTTLPR) have a larger response to negative emotional stimuli and higher spontaneous activity during the resting state than non-carriers. However, recent studies have suggested that the effects of 5-HTTLPR may be specific to different ethnic groups. Few studies have been conducted to address this issue.

**Methodology/Principal Findings:**

Blood oxygenation level dependent (BOLD) functional magnetic resonance imaging (fMRI) was conducted on thirty-eight healthy Han Chinese subjects (l/l group, n = 19; s/s group, n = 19) during the resting state and during an emotional processing task. Compared with the s/s group, the l/l group showed significantly increased regional homogeneity or local synchronization in the right amygdala during the resting state (|*t*|>2.028, *p*<0.05, corrected), but no significant difference was found in the bilateral amygdala in response to negative stimuli in the emotional processing task.

**Conclusions/Significance:**

5-HTTLPR can alter the spontaneous activity of the amygdala in Han Chinese. However, the effect of 5-HTTLPR on the amygdala both in task state and resting state in Asian population was no similar with Caucasians. They suggest that the effect of 5-HTTLPR on the amygdala may be modulated by ethnic differences.

## Introduction

Previous studies have revealed a critical role of the serotonin (5-HT) neurotransmitter system in the development of emotional circuitry and the onset of affective disorders [1]–[4]. The serotonin transporter (5-HTT) plays a crucial role in the reuptake of serotonin at brain synapses and it has been widely recognized as the target site of selective serotonin reuptake inhibitors (SSRIs), which have been recognized as both anti-depressant and anti-anxiety drugs [5]. One common polymorphic variant of the 5-HTT-linked polymorphic region (5-HTTLPR, SLC6A4) is located on the chromosome 17q11.1–q12 [6] which is a variable repeat sequence resulting in two common alleles: the short (s) variant and the long (l) variant. The s allele of the 5-HTTLPR polymorphism, which affects the promoter of the 5-HTT gene, causes reduced uptake of the neurotransmitter serotonin into the pre-synaptic cells in the brain [3]. Many studies suggest that the s allele of 5-HTTLPR may confer genetic risk for affective disorder [7], such as major depressive disorder [8]–[10].

Advances in noninvasive functional neuroimaging techniques provide a unique opportunity to explore and evaluate the functional impact of brain-relevant genetic polymorphisms more rapidly and with greater sensitivity than existing behavioral assessments [11]. Several blood oxygenation level dependent (BOLD) functional magnetic resonance imaging (fMRI) studies have shown that 5-HTTLPR s carriers have higher evoked activity to negatively valenced stimuli [12]–[15] and higher resting-state cerebral blood flow (CBF) [16], [17] in the amygdala than non-carriers, suggesting a potential underlying mechanism by which the s allele confers affective disorder.

However, recent studies have suggested that the effects of 5-HTTLPR on amygdala activity may be specific to different ethnic groups [18], [19]. The genotype distribution is quite different in different ethnic populations, with the s/s allele prevalence around 12–24% in Caucasians but around 45–74% in East Asians [20]. In Asian populations, some studies found a significant association between the s allele and affective disorder [21], but more studies found no significant association [22]–[25] and one study even found that the l allele of 5-HTTLPR was more common in major depression disorder (MDD) group than in healthy controls [26]. A recent meta-analysis study also found that the s/s genotype was significantly associated with an increased risk of MDD among Caucasian population but not among Asians [10]. Ethnic differences were also reported in treatment response to SSRIs. Most studies support the association of the l/l genotype or l allele with treatment response in Caucasians [27]–[29], whereas most studies with Asian samples have shown contradictory results [22], [30], [31]. Such evidence has also come from fMRI studies. There have been only two studies [18], [19] that explored the association of 5-HTTLPR and amygdala activity in Asian populations. These studies showed higher activation in the amygdala of l carriers [18] or l/l homozygous subjects [19] than in s carriers, findings contrary to previous results in the Caucasian population [12]–[15], [32].

Most previous studies focused on the differences of task activation of the amygdala between different genotypes [12], [13], [15], [18]. However, activation is the signal change from baseline or control states to task states. Both the task and control states usually vary from study to study and the results are therefore less comparable between different studies. Resting-state brain function is drawing more and more attention. Recently, three studies have applied resting-state fMRI (RS-fMRI) to explore the relationship between the intrinsic activity of the amygdala and the 5-HTTLPR polymorphism in Caucasians [16], [17], [33]. Two of these studies found that s carriers had higher CBF than non-carriers [16], [17] and the third study failed to detect significant differences between different genotype groups [33]. This information may be critical in interpreting the different manifestations of responses to aversive stimuli in l and s allele carrier groups and have a very important role in helping us to understand the brain's ongoing activity between the occurrences of negative external stimuli [17]. However, all subjects of the three resting-state fMRI studies were Caucasians. Thus, whether the effects of the 5-HTTLPR on spontaneous activity in the amygdala have ethnic differences in Asian population should be investigated.

The current study aimed to investigate the effects of 5-HTTLPR on the spontaneous activity as well as evoked activity of the amygdala in Han Chinese. We hypothesized that the effect of 5-HTTLPR genotype on amygdala activity would be different in Han Chinese from Caucasian population. In addition, we were also interested in the effects of 5-HTTLPR on the functional connectivity of the amygdala with other brain areas.

## Results

### Allele distribution of the 5-HTTLPR gene in Han Chinese

The allele frequency of this polymorphism did not show any deviation from the Hardy-Weinberg equilibrium. The allele frequencies of the s and l alleles for the 5-HTTLPR in our sample of 663 healthy Han Chinese sample (72% and 28%, respectively) were significantly different from those of a Caucasian sample (42% and 58%, χ^2^ = 18.4, d.f. = 1, *p*<0.001) [34] but similar to the findings in other studies about Asian populations (79% and 21%) [24].

### Behavioral data

**Figure 1:**
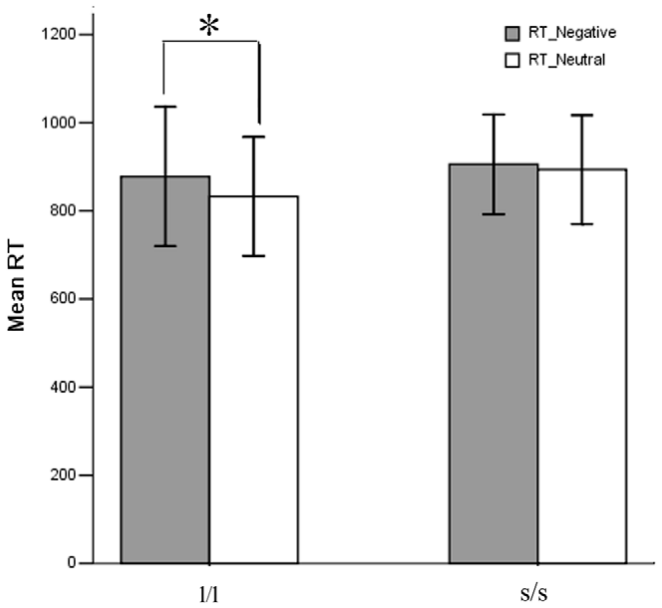
Difference in reaction time to negative stimuli and neutral stimuli in l/l group and s/s/ group. **, *p*<0.01.

There are 38 participants involved in this study, in which 19 were homozygous for the s allele (s/s group) and 19 were homozygous for the l allele (l/l group) of 5-HTTLPR. The demographic and behavioral data are listed in Table 1. There were no significant differences in age, gender, the score of Beck Depression Inventory II (BDI) [35], the score of Beck Anxiety Inventory (BAI) [36], and the score of harm avoidance of Temperament and Character Inventory-Revised (TCI-R) [37] between the two groups (all *p*s>0.05). Repeated measure analysis of variance (AVOVA) on the accuracy of the emotional task revealed no significant main effects and interaction effect (*p*s>0.05). ANOVA on the reaction time showed no significant between-group effect, but showed significant within-group effect (*F_1,36_* = 11.361, *p* = 0.002), i.e., slower response to negative (892±136 ms) stimuli than to neutral (863±131 ms). The reaction time showed a trend of significant interaction (*F_1,36_* = 3.903, *p* = 0.056). Post hoc paired *t*-tests showed that the slower reaction time for negative stimuli existed in l/l group (*t_18_* = 3.738, *p* = 0.002) but not in s/s group (*t_18_* = 0.998, *p* = 0.332) (Figure 1).

**Table 1:** Demographics and scores of behavioral measurements of the two genotype groups.

	s/s group (Mean ± SD)	l/l group (Mean ± SD)	*p*
Age	20.58±0.90	20.26±0.99	0.31
Male/Female	9/10	10/9	0.75
BDI	9.95±6.51	10.11±7.75	0.95
BAI	9.47±5.68	9.42±6.10	0.98
Harm avoidance	90.95±19.14	93.67±19.40	0.67^a^

BDI, Beck Depression Inventory. BAI, Beck Anxiety Inventory.

a *TCI score of a subject in the the l/l group was missing.*

### Imaging data during resting state

#### Results of regional homogeneity (ReHo) analysis

One-sample *t*-tests for the s/s group and the l/l group showed that the default mode network including the posterior cingulate cortex (PCC)/precuneus, medial prefrontal cortex (MPFC), and bilateral angular gyrus, exhibited significantly higher ReHo [38] than the global mean. This pattern was closely consistent with previous studies [38], [39].

**Figure 2:**
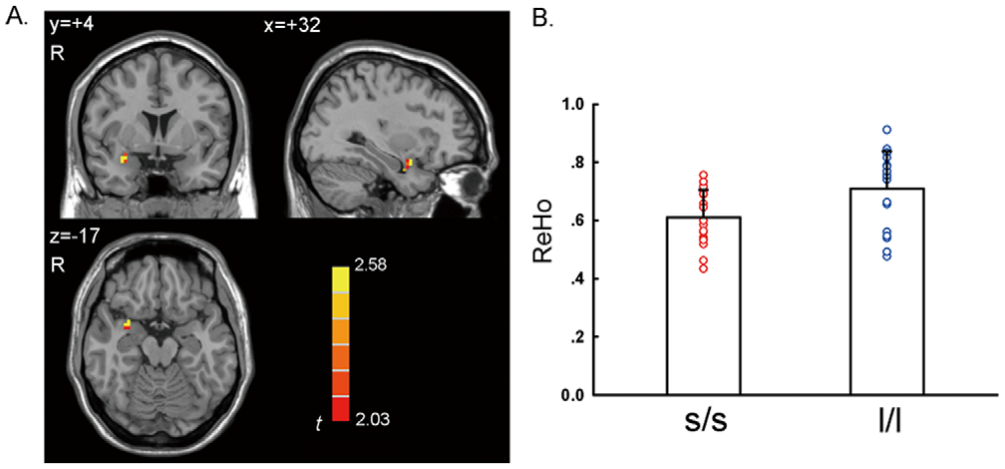
Significant higher ReHo in the right amygdala in l/l group than s/s group. A. Voxels with |*t*|>2.028 and cluster size >297 mm^3^ (11 voxels) corresponded with a corrected *p*<0.05. R: right side. B. Bars and error bars represent the mean and standard deviation of ReHo value in the right amygdala, which showed significant difference between the two genotype groups in Figure 1A.

**Figure 3:**
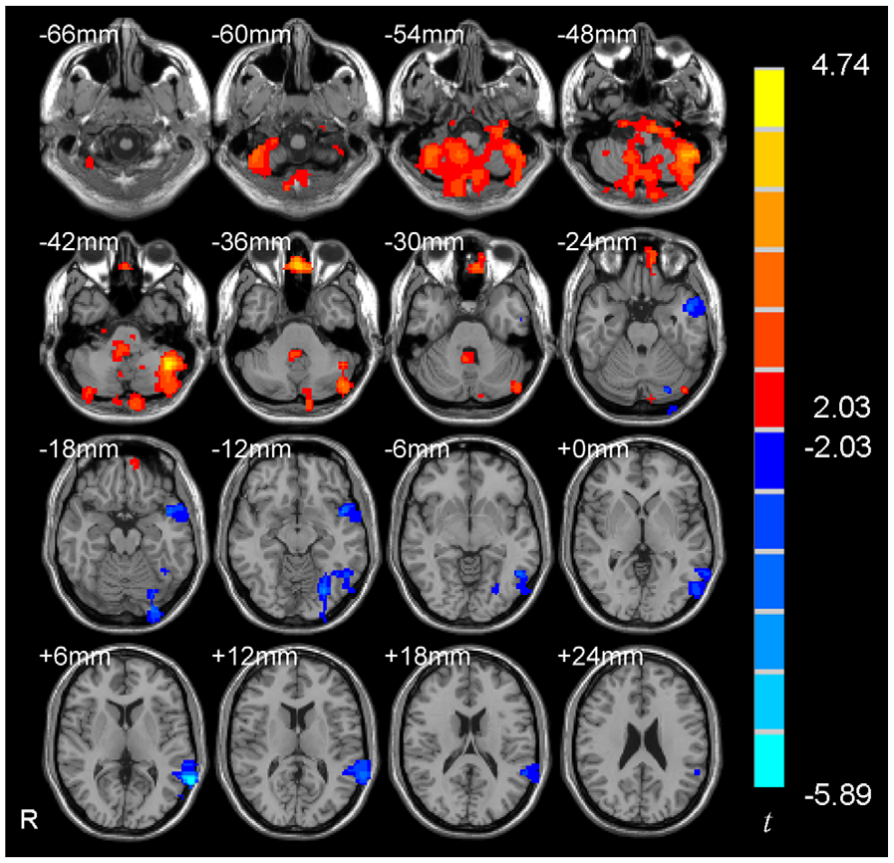
Significant different ReHo in s/s group than l/l group in a whole brain analysis. Voxels with |*t*|>2.03 and cluster size >6156 mm^3^ (228 voxels) corresponded with a corrected *p*<0.05. R: right side.

The two-sample *t*-test showed that the l/l group had significantly higher ReHo than the s/s group in the right amygdala (Figure 2 and Table 2, *p*<0.05, corrected) in a region of interest (ROI) analysis. There was no significant correlation between the ReHo value of the right amygdala and the score of behavioral measurements (BAI, BDI, and harm avoidance of TCI) (*p*s>0.05). In addition, a few other brain regions showed significant between-group difference, including cerebellum, superior temporal gyrus, and fusiform gyrus in a whole brain analysis (Figure 3 and Table 2, *p*<0.05, corrected in the whole brain).

**Table 2:** Significant difference of ReHo in the two genotype groups (s/s vs. l/l).

	Cluster size (Voxels)	Peak coordinates in MNI (x, y, z)	*t* value	*p* value
ROI analysis				
R amygdala	11	33, 3, −18	−2.58	0.014
Whole brain analysis				
L cerebellum	2959	−45, −54, −42	4.74	0.00003
L STG	253	−48, 9, −15	−4.15	0.0002
L FG	787	−69,−57,6	−5.89	0.000005

R: right. L: left. STG: superior temporal gyrus. FG: fusiform gyrus.

#### Results of between-group difference in functional connectivity of the amygdala

**Figure 4:**
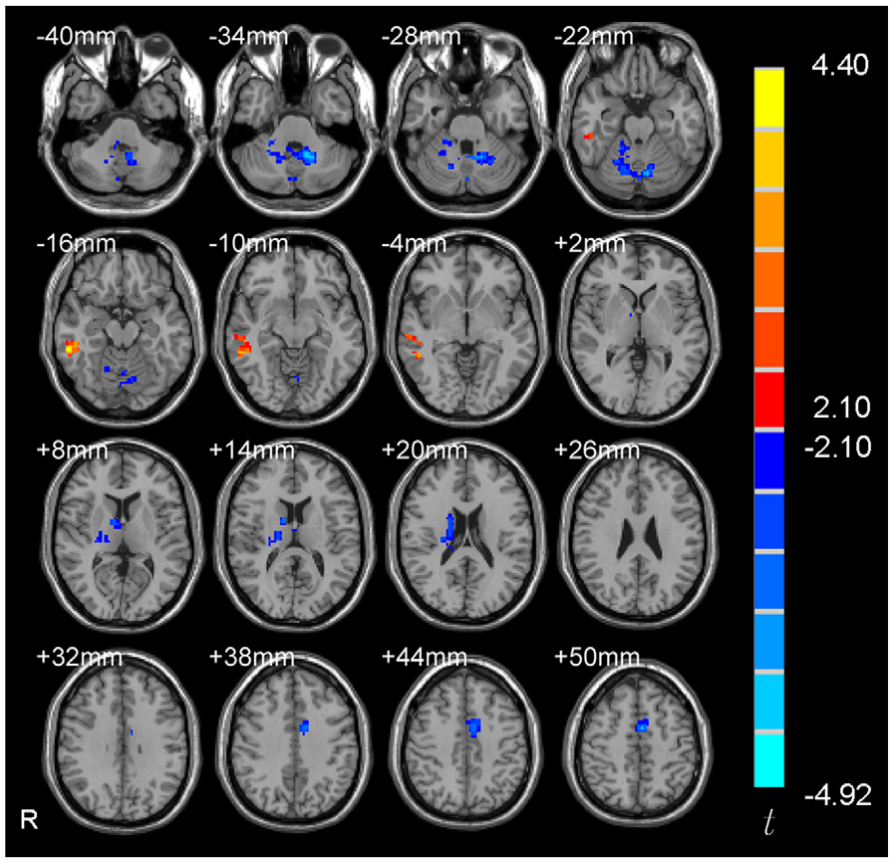
Significant between-group difference of the functional connectivity of the left amygdala between the two genotype groups, *p*<0.05, corrected, voxels with |*t*|>2.10 and cluster size >2835 mm^3^ (105 voxels). R denotes the right side of the brain.

**Figure 5:**
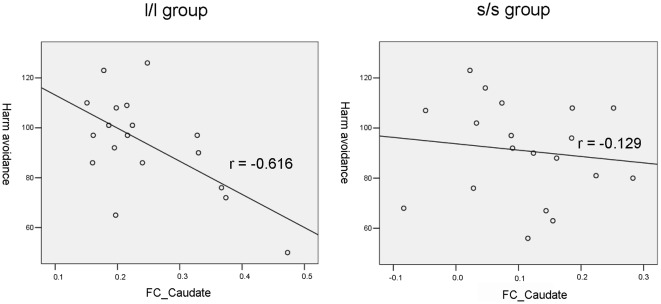
Correlation between harm avoidance score and the strength of functional connectivity between the left amygdala and right caudate in the two genotype groups. FC_Caudate: the strength of functional connectivity between left amygdala and right caudate in each group.

Some studies have investigated the 5-HTTLPR genotype effects on the functional connectivity of the amygdala during task states [14], [15]. However, the results were inconsistent. No resting-state amygdala functional connectivity study has been carried out to investigate the 5-HTTLPR genotype effects. Therefore, as an exploratory study, we compared the functional connectivity with amygdala between s/s and l/l group. Two-sample *t*-tests showed that no brain region had significant difference in functional connectivity with the right amygdala between the two groups. Whereas, four brain regions including right caudate, middle cingulate gyrus, middle temporal gyrus and cerebellum showed significant difference in functional connectivity with the left amygdala (Figure 4 and Table 3) (*p*<0.05, corrected). We further performed correlation analysis between the functional connectivity strength of the four brain regions and the behavioral data (the score of BAI, BDI and harm avoidance of TCI) for each group of participants. The right caudate showed significant negative correlation between the functional connectivity strength and the score of harm avoidance in l/l group (*r* = −0.616, *p* = 0.007) (Figure 5). No other significant correlation was found.

**Table 3:** Significant difference in functional connectivity with the left amygdala between the two genotype groups (s/s vs. l/l).

	Cluster size (Voxels)	Peak coordinates in MNI (x, y, z)	Mean *Z* in s/s	Mean *Z* in l/l	*T* value	*P* value
R MTG	129	57, −36, −12	0.019	−0.158*	3.30	0.004
R caudate	147	12, 0, 12	0.163*	0.323*	−3.58	0.002
L MCG	119	−3, 0, 51	0.016	0.203*	−4.01	0.001
L Cere	427	−15, −51, −30	−0.090	0.156*	−4.92	0.0001

R: right. L: left. MTG: middle temporal gyrus. MCG: middle cingulate gyrus. Cere: cerebellum.

* : showing significant functional connectivity by one-sample *t*-test within group.

### Imaging data in the emotional processing task

**Figure 6:**
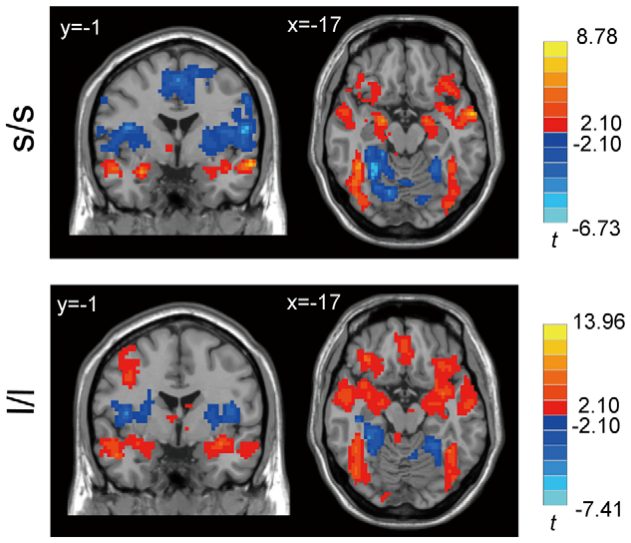
Activation in the negative condition versus the neutral condition in the two genotype groups. *p*<0.05, corrected, voxels with |*t*|>2.10 and cluster size >6156 mm^3^ (228 voxels).

In comparison to the neutral condition, the negative condition showed significantly higher activation in the bilateral amygdala in both the s/s group and the l/l group (Figure 6, *p*<0.05, corrected). In addition to the amygdala, there were significantly higher activation to negative stimuli in bilateral posterior fusiform and parahippocampal areas, the anterior cingulate cortex (ACC), and the ventral prefrontal cortex (vPFC). The activation patterns were consistent with previous studies [40].

**Figure 7:**
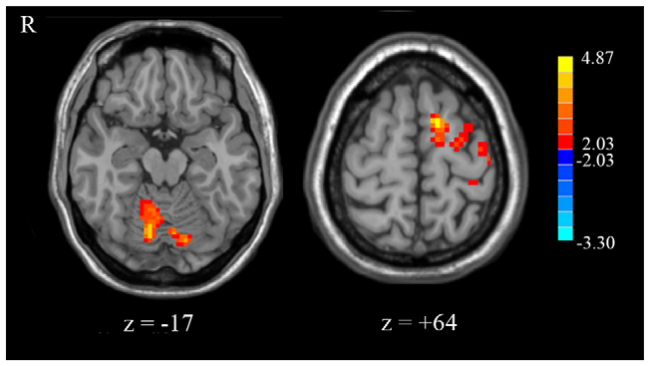
Significant difference in activation in l/l group and s/s group in a whole brain analysis. Voxels with |*t*|>2.03 and cluster size >6156 mm^3^ (228 voxels) corresponded with a corrected *p*<0.05. R: left side.

Direct comparison of the two groups didn't find significant different activation in the bilateral amygdala (*p*>0.05, corrected in the amygdala). But two voxels showed greater activation (*p*<0.05, Table 4) in the l/l group than in the s/s group without correction for multiple comparisons. In addition, right cerebellum and left supplementary motor area (SMA) showed significant higher activation in the l/l group than s/s group in an exploratory analysis (Figure 7 and Table 4, *p*<0.05, corrected within the whole brain).

**Table 4:** Significant difference in activation during the emotional task between the two genotype groups (l/l vs. s/s).

	Cluster size (Voxels)	Peak coordinates in MNI (x, y, z)	*t* value	*p* value
*ROI analysis* *				
L amygdala	2	−30, −3, −15	2.28	0.029
*Whole brain analysis* #				
R Cerebellum	472	12, −72, −18	3.70	0.0007
L SMA	215	−12, 6, 63	4.04	0.0003

* : (p<0.05, not corrected).

# : (p<0.05, corrected).

R: right. L: left. SMA: supplementary motor area.

## Discussion

The present study investigated the effects of 5-HTTLPR on amygdala activity both in an emotional processing task and in the resting state in Han Chinese. The result from the resting state demonstrated higher activity in the amygdala of the l/l group compared with that in the s/s group, but no significant difference was found in the emotional task between the two groups.

Studies of 5-HTTLPR genotype effects on emotional task activation have consistently reported higher activation in s/s or s carriers than l/l in Caucasian populations [12]–[15], [32]. The current study did not find significant difference in task activation in Han Chinese. When we used a liberal threshold (i.e., *p*<0.05 without multiple comparison correction), two voxels showed higher activation in l/l group than s/s group. Therefore, this result was not consistent with those from Caucasians. There has been so far only one research article about the genotype effects of 5-HTTLPR on amygdala activity in Asians [18]. In that study, it was found that the l carriers had higher activation in the bilateral amygdala than the s/s group during an angry task. In addition, a review reported a personal communication (SE Taylor, March 22, 2007) and mentioned greater amygdala activation in the l homozygous than in s carriers, most participants being East Asian ancestry [19]. The reaction time of emotional task in the current study provided interesting evidence of 5-HTTLPR genotype effects on emotional processing. The results of non-significant between-group difference was consistent with previous studies [12], [13]. However, these studies did not compare the difference between conditions. In the current study, ANOVA showed significant main effect of emotional stimuli on reaction time, i.e., a slower reaction time to negative stimuli than to neutral stimuli. This result was consistent with previous studies [41], [42], and suggesting that the emotional task requires more complicated neural processing. Our further analysis showed that such slower reaction time to negative stimuli existed in l/l group but not s/s group. The current result may imply that different genotype of 5-HTTLPR may have different behavior performance during emotional processing. However, such effect is not strong enough (marginal interaction effect of *p* = 0.056) to draw a conclusion. Several studies had reported that s carriers of 5-HTTLPR exhibited a stronger attention bias for anxious stimuli than participants with l/l in Caucasians [43]–[45]. The results from Caucasians and the current result looks contradictory, suggesting that 5-HTTLPR might have ethnic effect. It will be interesting for future studies to further investigate this ethnic effect, especially involving both Caucasians and East Asian populations in a study.

We found higher ReHo in the right amygdala in the l/l group than s/s group. The physiological meaning of the ReHo difference in the right amygdala between l/l and s/s groups remains unclear. ReHo measures the local synchronization of BOLD signal. An electrophysiological study has shown high local synchronization between the local field potentials (LFPs) of multiple cortical electrodes with a physical distance of 2.6–10.6 mm and such local synchronization in resting state could be modulated by stimulation [46]. From this aspect, ReHo measurement of BOLD fMRI was similar with the local synchronization of LFPs of multiple cortical electrodes. ReHo has been used in many studies on brain disorders, of which, the results of two studies on epilepsy were interesting. Increased ReHo was found in the medial temporal lobe of patients with temporal lobe epilepsy (TLE) [47] and also found in the thalamus of patients with generalized tonic-clonic seizures (GTCS) [48]. These results were consistent with the hypothesis that the abnormally increased local synchronization of neural activity may underlie the epileptic discharges [47], [48]. Thus the current RS-fMRI result may suggest that the l/l group had higher synchronization of spontaneous neuronal activity in the right amygdala than the s/s group. However, this result of Han Chinese seems to be contrary to those from two RS-fMRI studies in Caucasian population [16], [17], which reported that s carriers or the s/s homogeneous group had higher CBF in the amygdala than the l/l group. These two studies used arterial spin labeling (ASL) fMRI technique and measured regional CBF while the current study used BOLD fMRI and measured the synchronization (i.e., ReHo) of BOLD signals. It has been reported that resting-state CBF and resting-state ReHo have had a high spatial correlation in the default mode network [49]. Of note, whether such high spatial correlation exists in the amygdala warrants further investigation as the amygdala was not spatially covered in that previous correlation study [49]. Anyway, it is less likely that the reversed results of the current study on Asians compared with that of previous studies on Caucasians were due to the different resting-state imaging techniques (i.e., CBF vs. BOLD). A more reasonable interpretation would be ethnic differences of the effects of the 5-HTTLPR polymorphism.

The right amygdala showed no significant difference between the two groups in functional connectivity. Significant differences in functional connectivity of the left amygdala were found in the right caudate, middle cingulate gyrus, middle temporal gyrus and cerebellum between the two groups. Previous amygdala functional connectivity studies on 5-HTTLPR genotype effects focused on task state and investigated functional connectivity between two predefined regions, e.g., between the amygdala and ventral medial prefrontal cortex [14] and between the amygdala and anterior cingulate cortex [15]. All these four brain regions in the current study had not been reported in previous amygdala functional connectivity studies. An interesting finding in the current study is the higher positive functional connectivity between the right caudate and left amygdala in the l/l group than s/s group. This connectivity strength showed significantly negative correlation with harm avoidance score of TCI in l/l group but not in s/s group. An RS-fMRI study has demonstrated increased connectivity between the amygdala and caudate in patients with schizophrenia [50] and a task fMRI study found increased connectivity between the amygdala and the caudate during masked fear condition in borderline personality disorders [51]. However, the existing evidence of the amygdala functional connectivity are not enough to draw a conclusion.

Previous studies showed that 5-HTTLPR can alter amygdala activity in response to salient stimuli but that the direction of this association may be influenced by genetic background (i.e., ethnicity) [19]. The results of both task state and resting state from the current study provide new evidence for this hypothesis. The current study may explain why the Asian population has a higher frequency of the s allele but a lower prevalence of affective disorders compared with Caucasian population [20]. Ethnic differences may be important in determining disease risk factors and optimizing treatment [52].

There were several limitations in the present study. First, the result of no significant difference between the two genotype groups in the emotional processing task may be due to either smaller sample size or ill-designed task. Genetic effects on affective disorder is complex. The impact of one particular gene polymorphism on brain function may be very small and therefore the sample size should be large enough to detect statistical significance [53], [54]. The task design could be further optimized. For example, a previous study in Korean women reported higher amygdala activation in l carriers than s/s group, but only for angry emotion and not sad emotion [18]. The negative scene pictures in the current study might have induced complex emotion responses. In addition, we used only negative and neutral stimuli whereas positive stimuli may provide additional information. Therefore, future studies with a well designed task, larger sample size, and even a combination of BOLD and CBF will clarify the genotype difference. Second, we didn't include a Caucasian population sample and perform the same procedures to elucidate any ethnic differences of genotype effect. Future studies should be conducted on two population samples, i.e. Caucasians and Asians, about the 5-HTTLPR effect on amygdala activity. Third, our genotyping of the 5-HTTLPR did not differentiate an uncommon alleles (xl) that are longer than the l variant and we did not analyze the recently reported A to G single nucleotide polymorphism (SNP) within the long allele [55], [56]. The role of xl is unknown. The l_G_ allele was found to behave similarly to the lower-expressing s allele [57], and it can increase amygdala activation more than the l_A_ allele [58], [59]. Previous studies have reported that the frequency of xl [20] and l_G_ [60] allele was relatively low in Asians. To exclude these confounding factors would be helpful to reveal the ethnic effect of the genotypes of 5-HTTLPR in the future studies.

In conclusion, our study suggested that 5-HTTLPR can alter the spontaneous activity of the amygdala in Han Chinese and the association between the 5-HTTLPR polymorphism and the activity of the amygdala may be modified by ethnic differences both in response to negative stimuli and during the spontaneous activity of the resting state. Further investigation of both Caucasian and East Asian populations and more detailed genotyping (e.g., A to G SNP within the long allele) will help to clarify this issue.

## Methods and Materials

### Participants

This study was approved by the Ethics Committee of the Key Laboratory of Cognitive Neuroscience and Learning, Beijing Normal University. There were two stages of informed consent. For the first stage, blood samples were obtained from 663 volunteers who were healthy freshman at Beijing Normal University when they were taking a physical examination. All participants gave written informed consent in which they were told that the genotyping results would not be given to the participants. At the second stage, 42 right-handed participants (mean age 20.4 years, range 18–22 years) were selected for MRI scanning according to genotype (See below) after they gave written informed consent. But they were not aware of their genotypes. None of them had reported any history of psychiatric or neurological illness. Two participants were excluded due to excessive head motion and two participants were excluded due to falling asleep during the resting-state fMRI scanning (See data analysis for details). For the remaining 38 participants, 19 were (9 males) homozygous for the s allele in the s/s group and 19 were (10 males) homozygous for the l allele in the l/l group. Participants in the two genetic groups were similar in age (*p* = 0.31, two-sample *t*-test) and gender (*p* = 0.75, chi-square test).

### Mood and personality assessment

The BDI, BAI, and TCI-R were used as behavioral measures of depressive symptoms, anxiety symptoms, and personality dimensions, respectively.

### RS-fMRI scanning

During the RS-fMRI session, the participants were instructed to keep as still as possible and not to think systematically. Two subjects were excluded due to fall asleep during the scanning. All other subjects reported that they had never fallen asleep during the RS-fMRI scanning.

### Emotional task fMRI scanning

During the task-state fMRI scanning, 60 pictures (30 negative and 30 neutral, 30 indoor and 30 outdoor) were chosen from the International Affective Picture System (IAPS) [61]. The negative pictures depict complex unpleasant events including pollution, starving children, and cemeteries [61]. Previous study reported a high correlation (*r* = 0.913, *p*<0.01) of the valence scores of IAPS pictures between Han Chinese and Caucasians [62]. Each picture was presented for 1500 ms. The participant was instructed to press the left or right button to judge whether the picture was indoor or outdoor as quickly as possible. Then, a fixation “+” was presented for 500 ms to 6500 ms (randomly jittered between 500, 2500, 4500 and 6500 ms). The order of the categories of pictures (neutral and positive, indoor and outdoor) was randomized. The emotional processing task lasted 5 min 2 s.

### MRI data parameters

MR images were collected using a SIEMENS TRIO 3-Tesla in the Brain Imaging Center for brain research, Beijing Normal University. Participants lay supine with head snugly fixed by belt and foam pads to minimize head motion. Each participant underwent an eight-minute RS-fMRI scanning session, an emotional processing task-state fMRI scanning session and a 3D anatomic session. The functional images were obtained with the following parameters: 33 axial slices, thickness/gap = 3/0.6 mm, in-plane resolution = 64×64, repetition time (TR) = 2000 ms, echo time (TE) = 30 ms, flip angle = 90°, field of view (FOV) = 200×200 mm^2^. The 3D T1-weighted magnetization-prepared rapid gradient echo (MPRAGE) image was acquired with the following parameters: 128 sagittal slices, slice thickness/gap = 1.33/0 mm, in-plane resolution = 256×192, TR = 2530 ms, TE = 3.39 ms, inversion time (T1) = 1100 ms, flip angle = 7°, FOV = 256×256 mm^2^.

### RS-fMRI data analysis

#### Preprocessing

Statistical Parametric Mapping 8 (SPM8, http://www.fil.ion.ucl.ac.uk/spm) and Data Processing Assistant for Resting-State fMRI (DPARSF, http://www.restfmri.net) [63] were used to preprocess the fMRI data. The first 10 volumes of the functional images were discarded to allow participants to get used to the fMRI scanning noise. Slice timing and head motion correction were conducted. For the RS-fMRI scanning, no participant had head motion more than 2.0 mm of displacement or 2.0° of rotation throughout the course of scan. The individual 3D image was coregistered to the mean functional image after motion correction using a linear transformation. The transformed 3D images were then segmented into grey matter, white matter and cerebrospinal fluid by the unified segmentation approach. Normalization parameters were applied to the coregistered functional images and resliced to 3 mm isotropic resolution.

#### ReHo analysis

Resting-State fMRI Data Analysis Toolkit (REST, www.restfmri.net) [64] was then used for linear trend removing, temporally band-pass filtering (0.01∼0.08 Hz) [57], [65] and ReHo computation. ReHo used the Kendall coefficient of concordance (KCC) [66] to measure the temporal synchronization of the time series within a functional cluster. This method had been widely used in clinical studies by several different groups [67]–[74]. In the current study, 27 nearest neighboring voxels were defined as a cluster and a KCC value (range 0–1) was given to the voxel at the center of this cluster. Then the ReHo maps were spatially smoothed (FWHM = 6 mm) using SPM8. Each individual ReHo map was then standardized by dividing its mean ReHo of the entire brain [38]. One-sample *t*-tests against one were conducted on the ReHo maps for the two genotype groups separately to show the general patterns of resting-state spontaneous activity. A two-sample *t*-test was performed to compare the two genotype groups within the bilateral amygdala. The amygdala ROIs were predefined by the anatomical automatic labeling (AAL) template [75]. Because we had a strong *a priori* hypothesis regarding differential activity in the amygdala, we used a small volume correction for multiple comparisons within the amygdala ROIs and set corrected *p*<0.05 as the threshold (individual *p*<0.05, cluster size >11 voxels/297 mm^3^). The statistical result was corrected for multiple comparisons using the “AlphaSim” implemented in REST. This function is based on the Monte Carlo simulation in AFNI (http://afni.nih.gov/afni/docpdf/AlphaSim.pdf). After the ROI analysis, an exploratory investigation was performed in the whole brain to reveal potential between-group difference in other brain regions. Voxels with *p*<0.05 and cluster size >6156 mm^3^ were considered significant, which corresponds to corrected *p*<0.05 as determined by AlphaSim in AFNI.

#### Functional connectivity analysis

A spherical (12 mm in diameter) seed ROI at the center of the right (x, y, z = +24, −4, −12) and left (x, y, z = −24, −4, −12) amygdala, respectively, was defined as similarly done in a previous study [14]. For each ROI, a seed reference timecourse was obtained by averaging the timecourses of all voxels in the ROI. Then Pearson's correlation analysis was performed between the seed reference timecourse and that of each voxel in the brain in a voxel-wise way. The 6 head motion parameters, the global mean timecourse, mean timecourse of the white matter, and mean timecouse of the cerebrospinal fluid were taken as nuisance covariates. Finally, the Fisher's z transformation was used to improve the normality of the correlation coefficients [57]. The above procedures were complemented by using REST [64]. Two-sample *t*-tests were performed between the two groups on the left and right amygdala functional connectivity maps, respectively. To constrain the comparison within the voxels showing significant functional connectivity with the amygdala, two masks (left-amygdala and right-amygdala mask, respectively) were generated by using one-sample *t*-tests. One-sample *t*-tests were conducted on the individual *z*-maps within each group and with each amygdala as seed. Voxels with *p*<0.05 and cluster size >6156 mm^3^ (corresponding to corrected *p*<0.05 as determined by AlphaSim in AFNI (http://afni.nih.gov/afni/docpdf/AlphaSim.pdf)) were considered significant. For the left amygdala functional connectivity map, voxels showing significant functional connectivity in either s/s group or l/l group formed the left-amygdala mask. The right-amygdala mask was generated in the same way. Multiple comparison correction for the two-sample *t*-tests was carried out in the left- and right-amygdala mask, respectively. For the left-amygdala mask, voxels with *p*<0.05 and cluster size >2835 mm^3^ (corresponding to corrected *p*<0.05) as determined by AlphaSim in AFNI were considered significant. For the right-amygdala mask, voxels with *p*<0.05 and cluster size >2754 mm^3^ (corresponding to corrected *p*<0.05) as determined by AlphaSim in AFNI were considered significant. Then, the Pearson's correlation analyses were implemented in SPSS 13.0 software (SPSS Inc., Chicago, Illinois, USA) to assess the association between behavioral scores (BAI, BDI, and harm avoidance of TCI, respectively) and the strength of functional connectivity with the amygdala in brain regions showing significant difference between two groups. The strength value of functional connectivity with the amygdala was derived from averaging the *z* value of all voxels in each region which showing significant functional connectivity difference between the two groups.

### Task fMRI data analysis

Preprocessing of the task fMRI data, including slice timing, head motion correction, and spatial normalization, were the same as for the RS-fMRI data except for not removing the first 10 time points. In the task-state fMRI scanning, two participants were excluded for further analysis due to head motion of more than 2.0 mm or 2.0°. Predetermined condition effects at each voxel were calculated using a *t*-test, producing a statistical image for the contrast of the negative condition versus the neutral condition for each participant. One-sample *t*-tests against zero were conducted on the individual contrast maps of the two genotype groups separately to show the general patterns of emotional task activation. Two-sample *t*-test was conducted on these contrast images to investigate task activation differences between the two genotype groups in the amygdala ROIs defined as in the above ReHo analysis. After the ROI analysis, an exploratory investigation was performed in the whole brain to reveal potential between-group difference in other brain regions. Voxels with *p*<0.05 and cluster size >6156 mm^3^ were considered significant, which corresponds to corrected *p*<0.05 as determined by AlphaSim in AFNI.

### Genotyping

Each participant provided 4 ml venous blood samples, and genomic DNA was isolated from blood samples using standard techniques. Forward (5′-GGC GTT GCC GCT CTG AAT TGC-3′) and reverse (5′-GAG GGA CTG AGC TGG ACA ACC AC-3′) primers were used to generate 484 bp and 528 bp fragments of 5-HTTLPR polymorphism. Polymerase chain reaction (PCR) was performed according to the protocol previously described [34] on a PE-9700 or a PE-2400 thermal cycle (Perkin Elmer, USA). After initial denaturation at 95°C for 4 min, 35 cycles were carried out at 96°C for 45 sec, 61°C for 90 sec, and 72°C for 90 sec, followed by a final step of elongation at 72°C for 10 min, then the PCR products were separated on a 2% agarose gel supplemented with ethidium bromide for 3 hr. They were then analyzed with Gel Doc 2000 imaging system to detect and record the genotype of each sample. The l variant represented the fragment of 528 bp and the s allele represented the fragment of 488 bp. Genotypes were read by at least two researchers, ambiguous or unidentifiable results were reamplified and re-scored. Samples that continued to amplify poorly were eliminated from the study.
